# Poncelet property and quasi-periodicity of the integrable Boltzmann system

**DOI:** 10.1007/s11005-020-01348-z

**Published:** 2021-02-01

**Authors:** Giovanni Felder

**Affiliations:** Department of Mathematics, ETH Zurich, 8092 Zurich, Switzerland

**Keywords:** Integrable dynamical systems, Poncelet theorem, Elliptic curves, 37J70, 14H70

## Abstract

We study the motion of a particle in a plane subject to an attractive central force with inverse-square law on one side of a wall at which it is reflected elastically. This model is a special case of a class of systems considered by Boltzmann which was recently shown by Gallavotti and Jauslin to admit a second integral of motion additionally to the energy. By recording the subsequent positions and momenta of the particle as it hits the wall, we obtain a three-dimensional discrete-time dynamical system. We show that this system has the Poncelet property: If for given generic values of the integrals one orbit is periodic, then all orbits for these values are periodic and have the same period. The reason for this is the same as in the case of the Poncelet theorem: The generic level set of the integrals of motion is an elliptic curve, and the Poincaré map is the composition of two involutions with fixed points and is thus the translation by a fixed element. Another consequence of our result is the proof of a conjecture of Gallavotti and Jauslin on the quasi-periodicity of the integrable Boltzmann system, implying the applicability of KAM perturbation theory to the Boltzmann system with weak centrifugal force.

## Introduction

In a 1868 paper with the unpretentious^1^ title “Solution of a mechanical problem” [1], L. Boltzmann, in his search of candidate dynamical systems obeying his Ergodic Hypothesis, introduced and studied a simple mechanical system. It describes of a particle moving in the region of a plane on one side of a straight line (the wall) and subject to a central force whose centre is not on the wall. When the particle hits the wall, it is reflected elastically. The force considered by Boltzmann is the sum of an attractive one with inverse-square law and a centrifugal force with inverse-cube law. We refer to [6, Appendix D] for an account of Boltzmann’s paper and of its significance for the evolution of statistical mechanics.

**Fig. 1:**
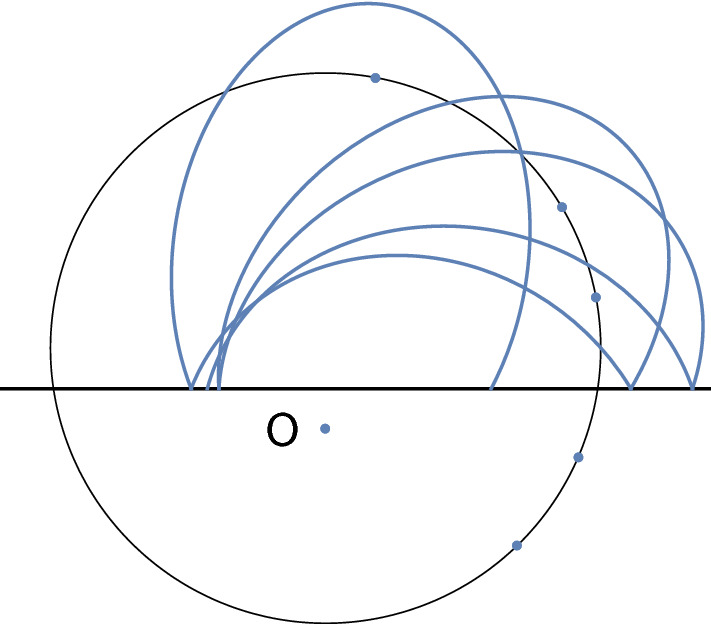
Trajectory of a particle subject to an attractive inverse-square law force bouncing off a wall (the horizontal line). The particle moves along elliptic arcs with one focus in the centre *O* and whose second focus lies on a circle

As first conjectured by G. Gallavotti [6, Appendix D], and proved by him and I. Jauslin [7], the system with pure inverse-square law has, additionally to the energy, a second independent integral of motion and is thus far from being ergodic. One way to express the existence of this independent integral is that the particle moves on arcs of Kepler trajectories with one focus at the centre and the second focus on a fixed circle; see Fig. 1 and Theorem 1. It was thus prudent of Boltzmann to add the centrifugal term.

It is convenient to describe the Boltzmann system as a discrete-time dynamical system by recording the point in phase space at each collision. The map sending a point to the point at the next collision is called the Poincaré map, and the orbits are obtained by iterating the Poincaré map.

**Fig. 2:**
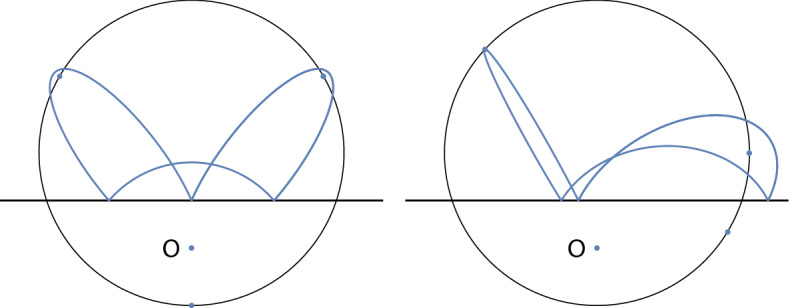
Two periodic orbits with integrals $D=7/4$, $E=-5/24$ in natural units

In this paper, we focus on this integrable case of the Boltzmann system, with zero centrifugal force, and we show that it has the *Poncelet property*: For given values of the two integrals of motion, either there are no periodic orbits or all orbits are periodic. See Fig. 2 for an example with period 3.

We call this the Poncelet property since it is shared by the discrete-time dynamical system underlying the Poncelet problem. Recall that the Poncelet problem asks for which pairs of ellipses there is a polygon inscribed in one and circumscribing the other. Poncelet’s theorem [15, Art. 570] states that if there is such a polygon for a given pair of ellipses, then there are polygons with a vertex at an arbitrary point of the circumscribing ellipse. Moreover, all these polygons have the same number of sides. See [2, 3] for a historical account and [4] for a review of recent developments.

A beautiful explanation of the Poncelet theorem was given by Ph. Griffiths [10] and Griffiths and J. Harris [8, 9], expanding on the classical work of Jacobi [13]. Consider the space of pairs (*P*, *a*) where *a* is a tangent line to the inner ellipse and $P\in a$ is a point of intersection of the tangent with the outer ellipse. On this space, we have two natural involutions: *i* maps (*P*, *a*) to $(P^{\prime },a)$ where $P^{\prime }$ is the other point of intersection and *j* maps (*P*, *a*) to $(P,a^{\prime })$ where $a^{\prime }$ is the other tangent through *P*. Taking iterates *x*, *t*(*x*), $t^2(x)$, $\ldots$ of the composition $t=j\circ i$ we obtain a broken line consisting of chords of the outer ellipse that are tangent to the inner one. And a polygon is formed if and only if $t^p(x)=x$ for some positive *p*. The observation of Griffiths and Harris is that the (complexified) space of such pairs is naturally a curve of genus one, and thus carries a free transitive action of its Jacobian, an elliptic curve. It is a general fact that if we have two non-trivial involutive automorphisms of a curve of genus one, both having fixed points, then their composition is the translation by an element of the elliptic curve. There are periodic orbits if and only if this element has finite order, in which case all orbits are periodic with the same period.

Our observation is that the integrable Boltzmann system behaves very much in the same way: we consider for fixed generic values of the two integrals of motion the pairs (*P*, *K*) consisting of a Kepler conic *K* and a intersection point $P\in K$ with the wall. We show that this space (after complexification and throwing in a couple of points at infinity) is a smooth curve of genus one carrying two involutions. The first keeps the Kepler conic and changes *P* to the other point of intersection with the wall and the second changes the conic to the other conic through *P* with the same integrals, obtained by elastic reflection of the particle at the wall. The orbits of the Boltzmann system are obtained by iterating the composition $t=j\circ i$ of these involution, implying the Poncelet property.

Another consequence of this observation is the proof of a conjecture of Gallavotti and Jauslin [7] that the motion is quasi-periodic for generic values of the integrals, namely that on generic level sets of the integrals there is an *angle variable*, a map to the circle, whose value increases by a fixed amount $\alpha$ at each iteration of *t*. This amount, which depends on the values of the energy and the second integral *D*, is a non-constant function of *D*. As discussed in [6, 7], this property, together with Boltzmann’s result that the map *t* preserves an area form on the level sets of the energy even in the presence of the centrifugal term, allows one to apply the Kolmogorov–Arnold–Moser perturbation theory. In this setting, the relevant theorem is Moser’s twist theorem [14] implying that for small centrifugal term most of the invariant circles on each energy surface are deformed to invariant circles. Thus one would need a sufficiently large centrifugal term to hope for an ergodic system.

Here we prove that the generic level sets of the integrals of motion is diffeomorphic either to a circle or to a pair of disjoint circles. The map *t* is indeed mapped to a translation by an amount $\alpha (D,E)$ whose *D*-derivative is generically non-vanishing, possibly composed with an involution exchanging the two connected components in the two-component case. This implies both Conjecture 1 and 2 in [7]. In loc. cit., a conjectural formula for the angle variable is given. It should also be possible to check that conjecture with our explicit formulae.

The quasi-periodicity and the distinction between the cases of one and two components arise from the classical theory of real elliptic curves of Abel, Jacobi,$\ldots$; see [5]. The level set $X_{{\mathbb {R}}}(D,E)$ is the set of real points of a smooth curve of genus one defined over ${\mathbb {R}}$ on which (if non-empty) we have a free and transitive action of the real points of an elliptic curve which is a Lie group isomorphic to the circle group $S^1={\mathbb {R}}/{\mathbb {Z}}$ (unipartite case) or $S^1\times {\mathbb {Z}}/2{\mathbb {Z}}$ (bipartite case).

In the unipartite case, the system is (periodic or) quasi-periodic, namely there is a diffeomorphism $\varphi :S^1\rightarrow X_{{\mathbb {R}}}(D,E)$ so that $\varphi ^{-1}\circ t\circ \varphi$ is a translation $\theta \mapsto \theta +\alpha (D,E)$ of the angle. The diffeomorphism can be given explicitly in terms of elliptic functions, see Theorem 4. In the bipartite case, the same holds for each component if we replace the map by its iterate $t^2$. The map *t* itself is given by the translation by an element of the Lie group $S^1\times {\mathbb {Z}}/2{\mathbb {Z}}$. If this element is not in the connected component of the identity, the orbits jump back and forth between the connected components. See Fig. 5 for a picture.

The transition between the unipartite and bipartite case happens when we cross a curve in the space of parameters ($D=2$ and $D=-2$ in our convention) where the elliptic curve degenerates to a nodal rational curve. We show that the unipartite case arises if $|D|<2$. If $D>2$ the real locus of the elliptic curve is bipartite and the action of *t* is by an element which is not in the identity component. If $D<-2$, which happens only for sufficiently large positive energy, the action is by an element in the identity component. Geometrically, we can understand the distinction from the constraint that the distance between foci is smaller than the major axis, which is determined by the energy. If $|D|>2$, all points on the circle on which the second focus moves satisfy this constraint, but if $|D|<2$, the motion is confined to an arc of this circle.

In the next section, we introduce the integrable case of the Boltzmann system, state the main result and deduce the Poncelet property. We also give an explicit example of a rational level set for which all orbits have period 3. The family of elliptic curve is constructed in Sect. 3 and we discuss the real locus and the quasi-periodicity in Sect. 4.

Recalling Boris Dubrovin’s recommendation that we should not forget that mathematics does not only consist of abstract theorems but also of calculations, we give explicit formulas for the diffeomorphism $\varphi$ and the rotation number $\alpha$.

## The integrable Boltzmann system

Let a particle subject to an attractive central force with inverse-square law move in a plane on one side of a straight line (the wall) not passing through the centre *O*. When the particle hits the wall it is reflected elastically.

To describe the system, we record the position and momentum of the particle each time it leaves the wall after a collision. We obtain a three dimensional discrete-time dynamical system given by iterations of the Poincaré map *t* sending the position and the momentum of the particle as it leaves the wall to the position and momentum of the particle after the next collision. We disregard the time it takes to go from one collision to the next.

For clarity of exposition, let us assume for the further discussion that the energy is negative and that the particle moves on the side of the wall not containing the center as in Fig. 1. Then the particle travels on arcs of ellipses with a focus in *O*, all with the same major axis. Our discussion holds also for the case of zero or positive energy, but one should place oneself in the projective plane and allow the particle to wander on hyperbolae to infinity and hit the wall from both sides.

Geometrically, we can think of the Poincaré map as a map on pairs (*P*, *K*), where *K* is an ellipse in the plane of motion with a focus in *O* and $P\in {\mathcal {K}}$ is an intersection point with the wall. The particle at *P* leaves the wall and follows the Kepler trajectory *K* until it reaches the other point of intersection $P^{\prime }$. At this point, the momentum is reflected and the particle continues on a new ellipse $K^{\prime }$ and $t(P,K)=(P^{\prime },K^{\prime })$. Thus the Poincaré map is the composition $t=j\circ i$ of two maps

$$\begin{aligned} i:(P,K)\mapsto (P^{\prime },K), \end{aligned}$$

exchanging the points of intersections of the ellipse with the wall and

$$\begin{aligned} j:(P^{\prime },K)\mapsto (P^{\prime },K^{\prime }), \end{aligned}$$

mapping a Kepler trajectory *K* to the trajectory $K^{\prime }$ whose momentum at $P^{\prime }$ is reflected at the wall.

The obvious fact that *i* and *j* are involutions will be important later.

We thus obtain a discrete dynamical system $(X_{{\mathbb {R}}},t)$, which we call the integrable Boltzmann system, on the three-dimensional configuration space $X_{{\mathbb {R}}}$ of pairs (*P*, *K*). The maps *i*, *j* make sense as birational maps in the complex domain of pairs (*P*, *K*) where *K* is a smooth conic in the two-dimensional affine space and *P* is an intersection point with an affine line. We thus get a complexification (*X*, *t*) where $t:X\dashrightarrow X$ is a birational map.

The next observation is that the Poincaré map preserves a second integral of motion in addition to the energy.

### Theorem 1

(Gallavotti–Jauslin [7]) The Poincaré map of the integrable Boltzmann system for a particle of mass *m* and a wall at distance *h* to the center preserves the energy *E* and the combination $D=L^2-2 h A_\perp$ of the angular momentum *L* and the component $A_\perp$ of the Laplace–Runge–Lenz vector perpendicular to the wall.

Recall that the (Hermann–Bernoulli–) Laplace–Runge–Lenz vector is the conserved quantity ${\mathbf {A}}={\mathbf {p}}\times {\mathbf {L}}-m\kappa \, {\mathbf {x}}/|{\mathbf {x}}|$ of the Kepler problem with Hamiltonian $|{\mathbf {p}}|^2/2m-\kappa /|{\mathbf {x}}|$. In terms of trajectories ${\mathbf {A}}$ is a vector in the plane of motion along the major axis whose length is the eccentricity times $m\kappa$.

Note that *i* preserves all conserved quantities $E,{\mathbf {L}},{\mathbf {A}}$ of the Kepler problem. Therefore, in fact,, both involution *i* and *j* preserve *E* and *D*.

We also observe that because of the classical relation $2mEL^2=|{\mathbf {A}}|^2-m^2\kappa ^2$ between conserved quantities of the Kepler problem, *D* can be written as

$$\begin{aligned} D=\frac{|{\mathbf {A}}|^2}{2mE}-2h\, A_\perp -\frac{m\kappa ^2}{2E}, \end{aligned}$$

so that the Laplace–Runge–Lenz vector moves on a circle of radius

$$\begin{aligned} R=\sqrt{m^2\kappa ^2+2mDE+4h^2 m^2E^2}. \end{aligned}$$

More geometrically, noticing that ${\mathbf {A}}/mE$ is the vector connecting the centre to the other focus, we can say that the particle moves along arcs of Kepler ellipses whose second focus lies on a circle of radius *R*/*m*|*E*| centeered at the mirror image of the centre with respect to the wall. 2

From now on, we choose for convenience units of time, length and mass so that $\kappa =1$, $h=1$, $m=1$.

The theorem is reduced in [7] to a geometric theorem on ellipses. Here is an alternative, possibly more direct proof. We can assume that the motion takes place in the plane with coordinates $x_1,x_2$ with the center at the origin and the wall at $x_2=1$. The phase space has coordinates $x_1,x_2,p_1,p_2$ and the integrals of motion of the Kepler problem are

$$\begin{aligned} E=\frac{p_1^2+p_2^2}{2}-\frac{1}{r}, \quad L=x_1p_2-x_2p_1, \quad A_1=p_2L-\frac{x_1}{r}, \quad A_2=-p_1L-\frac{x_2}{r}. \end{aligned}$$

Here $r=\sqrt{x_1^2+x_2^2}$ is the distance to the centre. In these coordinates,

$$\begin{aligned} D=L^2-2A_2=\frac{A_1^2+A_2^2-1}{2E}-2A_2. \end{aligned}$$

When the particle hits the wall at a point with $x_2=1$, the sign of $p_2$ is changed. Thus the angular momentum *L* and the orthogonal component $A_2$ of the Laplace–Runge–Lenz vector change to $L^{\prime }=-L-2p_1$, $A_2^{\prime }=-p_1L^{\prime }-1/r=A_2+2p_1L+2p_1^2$, respectively. Therefore $(L^{\prime })^2-2A_2^{\prime }=L^2-2A_2$, as claimed.

The complexified configuration space *X* is thus foliated by the level sets *X*(*D*, *E*) of the integrals of motion.

### Theorem 2

Let $D,E\in {\mathbb {C}}$ such that $D^2\ne 4$, $1+2ED+4E^2\ne 0$, $D+2E\ne 0$. Then the level set *X*(*D*, *E*) has a compactification ${\bar{X}}(D,E)$ which is a smooth projective curve of genus 1, and *i* and *j* extend to automorphisms with fixed points.

The construction of the compactification and the proof of this theorem is presented in the next section; see Theorem 3.

Thus we have a curve of genus one with two holomorphic involutions and we can reproduce the argument of Griffiths and Harris on the Poncelet theorem: Any smooth curve of genus one has a free transitive action of a group, the associated elliptic curve. The composition of two involutions with fixed points is the action an element of this group. More explicitly, by uniformization, we have an isomorphism

$$\begin{aligned} {\bar{X}}(D,E)\rightarrow {\mathbb {C}}/\varLambda , \end{aligned}$$

for some lattice $\varLambda ={\mathbb {Z}}\omega _1+{\mathbb {Z}}\omega _2$. Any holomorphic automorphism of ${\mathbb {C}}/\varLambda$ is of the form $z\mapsto az+b{\text {mod}}\varLambda$. An involution has $a=\pm 1$. A non-trivial involution with fixed points has $a=-1$. Thus the composition of two non-trivial involutions $z\mapsto -z+b$, $z\mapsto -z+c$ is the translation $z\mapsto z+v$ by $v=c-b\in {\mathbb {C}}/\varLambda$. This implies the following result.

### Corollary 1

Let $D,E\in {\mathbb {C}}$ satisfy the assumptions of Theorem 2. Then *t* is the action of an element of the associated elliptic curve. In particular, there is a biholomorphic map $\varphi :{\mathbb {C}}/\varLambda \rightarrow {\bar{X}}(D,E)$ for some lattice $\varLambda \subset {\mathbb {C}}$ such that $\varphi ^{-1}\circ t\circ \varphi (u)=u+T$ for all $u\in {\mathbb {C}}/\varLambda$ and some $T=T(D,E)\in {\mathbb {C}}/\varLambda$.

One consequence of this is the Poncelet property. The sequence $(t^n(x))_{n\in {\mathbb {Z}}}$ of images of $x\in {\bar{X}}(D,E)$ of iterates of *t* is called the orbit through $x\in {\bar{X}}(D,E)$. An orbit is called periodic if $t^p(x)=x$ (and thus $t^{n+p}(x)=t^n(x)$ for all *n*) for some positive integer *p*. The minimal such *p* is called the period of the orbit.

### Corollary 2

If for some *D*, *E* satisfying the assumptions of Theorem 2, the dynamical system $({\bar{X}}(D,E),t)$ has a periodic orbit, then all orbits in ${\bar{X}}(D,E)$ are periodic, and they all have the same period.

The orbits have period *p* if *T*(*D*, *E*) has order *p*, i.e., if *p* is minimal such that $p\,T(D,E)\equiv 0{\text {mod}}\varLambda$.

Thus we can determine the pairs (*D*, *E*) for which all orbits have period *p* by solving the equation $t^p(x)=x$ where $x\in X(D,E)$ is arbitrary. We get equations of countably many algebraic curves in the (*D*, *E*) plane for which all orbits are periodic. The case $p=1$ where *t* is the identity occurs in the degenerate range of parameter $D+2E=0$. In this case, all Kepler trajectories are tangent to the wall. An orbit of period $p=2$ corresponds to a conic intersecting the wall at right angle at both points of intersection. This arises only if $1+2ED+4E^2=0$, which is excluded by the theorem. In this degenerate case, the real locus $X_{{\mathbb {R}}}(D,E)$ consists of two points consisting of a conic which is symmetric with respect to the wall with its two intersection points. The first interesting case is $p=3$: if (*D*, *E*) lies on the algebraic curve

$$\begin{aligned} 4(D^2-4)E^2+4D(D^2-3)E+D^4-2D^2-3=0, \end{aligned}$$

for example if $D=7/4,E=-5/24$, all orbits in ${\bar{X}}(D,E)$ are periodic with period 3; see Fig. 2.

We conclude this section by discussing qualitatively the degenerate cases not covered by the theorem; see Theorem 3 for more detailed statements. As mentioned above, if $D+2E=0$ then ${\bar{X}}(D,E)$ consists of Kepler conics tangent to the wall. It is a rational curve. If $D=\pm 2$, the elliptic curve degenerates to a nodal curve with one node. The node is a fixed point corresponding to a degenerate conic (double line) orthogonal to the wall. The generic orbit $t^j(x)$ converges to it both for $j\rightarrow \infty$ and $j\rightarrow -\infty$. Finally, for $1+2ED+4E^2=0$, we get a nodal curve with two irreducible components and *t* maps one node to the other. The nodes represent a conic which is symmetric with respect to the wall with its two intersection points. In the latter two cases, the Poncelet property does not hold.

## A family of elliptic curves

We parametrize a point $(P,K)\in X(D,E)$ by the first coordinate *x* of $P=(x,1)$ and the Laplace–Runge–Lenz vector $(A_1,A_2)$. The corresponding Kepler trajectory (for $E<0$) is then determined by the properties that the the foci are (0, 0), $(A_1/E,A_2/E)$ and the major axis is $2a=-1/E$; see, e.g., [11, Section II]. From the condition that the sum of distances to the foci is 2*a* we deduce the equation of the Kepler ellipse

$$\begin{aligned} x_1^2+x_2^2=\left( L^2-A_1x_1-A_2x_2\right) ^2, \quad L^2=\frac{A_1^2+A_2^2-1}{2E}. \end{aligned}$$

Since $D=L^2-2A_2$, this can be written as

$$\begin{aligned} x_1^2+x_2^2=\left( 2A_2+D-A_1x_1-A_2x_2\right) ^2. \end{aligned}$$

This also holds for $E\ge 0$ with a similar derivation. Setting $x_2=1$ gives the equation for the two points of intersection of the Kepler trajectory with the wall.

Thus *X*(*D*, *E*) is defined as the algebraic set in the affine space with coordinates $(x,A_1,A_2)$ by the equations

1 $$\begin{aligned}&A_1^2+A_2^2-4EA_2=1+2DE, \end{aligned}$$

2 $$\begin{aligned}&x^2+1=(A_2+D-A_1x)^2. \end{aligned}$$

It will be useful, especially for the study of the real locus, to introduce the parameter $R^2=1+2DE+4E^2$, so that (1) describes a circle of radius *R*, and write (1) as

3 $$\begin{aligned} A_1^2+(A_2-2E)^2=R^2. \end{aligned}$$

The formulae for the involutions are

4 $$\begin{aligned} i(x,A_1,A_2)&=\left( -\frac{2(A_2+D)A_1}{1-A_1^2}-x,A_1,A_2\right) , \end{aligned}$$

5 $$\begin{aligned} j(x,A_1,A_2)&=(x,A_1^{\prime },A_2^{\prime }), \nonumber \\ A_1^{\prime }&=\frac{x^2-1}{x^2+1}A_1-\frac{2x}{x^2+1}A_2+\frac{4xE}{x^2+1},\nonumber \\ A_2^{\prime }&=-\frac{2x}{x^2+1}A_1-\frac{x^2-1}{x^2+1}A_2+\frac{4x^2E}{x^2+1}. \end{aligned}$$

The involution *i* exchanges the two solutions of (2). The second can be deduced from the calculation of how the Laplace–Runge–Lenz vector changes at a reflection, as in the proof of Theorem 2. We need to know two things from these formulae for the proof of Theorem 2:

- *i* and *j* are rational maps, i.e., maps that are defined on some dense open set and are given by rational functions of the coordinates. Since *i*, *j* are involutions (where defined), they are moreover birational maps, namely rational maps with rational inverses.
- Both have fixed points (in the complex domain). For *i*, they correspond to degenerate Kepler trajectories (double lines) and occur when the quadratic Eq. (2) for *x* has double roots. The fixed points of *j* are points $(x,A_1,A_2)$ so that the Kepler conic parametrized by $(A_1,A_2)$ meets the wall at *x* at a right angle. The values of the coordinates $(x,A_1,A_2)$ at the fixed point can be easily computed. For *i*, they are the solutions of (1) such that $A_2=-D/2$. For *j*, they are the solutions such that $A_2=2E/(1\pm R)$.

We turn to the description of the compactification of the space *X*(*D*, *E*) of solutions of (1), (2).

The first Eq. (1) describes a conic, which is smooth provided $R^2=1+2ED+4E^2\ne 0$. For each point in this conic, there are generically two solutions *x* of the second Eq. (2). It is useful to change variables by completing the square and setting $z=(1-A_1^2)x+A_1(A_2+D)$ so that the second equation becomes

6 $$\begin{aligned} z^2=A_1^2+(A_2+D)^2-1. \end{aligned}$$

Physically $z=\sqrt{D+2E}\,L$ is proportional to the angular momentum *L*.

In projective coordinates $A_1=\alpha _1/\alpha _0,A_2+D=\alpha _2/\alpha _0, z=\zeta /\alpha _0$, the equation are

7 $$\begin{aligned} \alpha _1^2+\alpha _2^2-2(D+2E)\alpha _0\alpha _2&=(1-(D+2E)D)\alpha _0^2, \end{aligned}$$

8 $$\begin{aligned} \zeta ^2&=\alpha _1^2+\alpha _2^2-\alpha _0^2. \end{aligned}$$

These equations define a family of curves in $\mathbb {CP}^3$ with projective coordinates $(\alpha _0:\alpha _1:\alpha _2:\zeta )$ over the affine plane with coordinates *D*, *E*. The fiber ${\bar{X}}(D,E)$ contains *X*(*D*, *E*) as an open dense subset. The map $p:(x,A_1,A_2)\mapsto (A_1,A_2)$ to the set of solution of (1) extends to a two-sheeted covering map $p:{\bar{X}}(D,E)\rightarrow C(D,E)$ to the plane curve defined by (7). It is the restriction to ${\bar{X}}(D,E)$ of the central projection $\mathbb {CP}^3\smallsetminus \{c\}\rightarrow \mathbb {CP}^2$ (a.k.a. the line bundle *O*(1)) with centre $c=(0:0:0:1)$ onto the plane $\zeta =0$.

The ramification locus is the intersection of (7) and the quadric $\alpha _1^2+\alpha _2^2-\alpha _0^2=0$ and consists generically of four points, so that ${\bar{X}}(D,E)$ is a generically a smooth projective curve of genus one.

If we exclude the line $D+2E=0$, where the two quadrics (7), (8) coincide, corresponding to the case where all Kepler trajectories are tangent to the wall, the number of ramification points is at most 4. They can be readily computed to be the points “at infinity” $P_\pm =(0:1:\pm \mathrm{i}:0)$ and

$$\begin{aligned} \left( 1:\pm \sqrt{1-\frac{D^2}{4}}:\frac{D}{2}:0\right) . \end{aligned}$$

The four ramification points are distinct if and only if $D\ne \pm 2$.

It will be useful to compute the action of *t* on the fixed points of *i* at infinity, which belong to all curves ${\bar{X}}(D,E)$.

### Lemma 1

Assume that $D+2E\ne 0$, and let $P_{\pm }=(0:1:\pm \mathrm {i}:0)\in {\bar{X}}(D,E)$. Then the image $Q_\pm =t(P_\pm )$ of $P_\pm$ under $t=j\circ i$ has projective coordinates

$$\begin{aligned} Q_\pm =\left( 1:\pm \mathrm {i}\,\left( -\frac{D}{2}+\frac{1}{2(D+2E)}\right) : \frac{D}{2}-\frac{1}{2(D+2E)}:\pm \mathrm {i}\right) . \end{aligned}$$

### Proof

Since $i(P_\pm )=P_\pm$, $t(P_\pm )=j(P_{\pm })$. A simple way to check the claim is to check that $Q_\pm$ belong to ${\bar{X}}(D,E)$, which is straightforward, and prove that $j(Q_\pm )=P_\pm$. By writing $Q_{\pm }=(1:A_1:A_2+D:z)$, we see that $A_1=\mp \mathrm{i}(A_2+D)$ so the relation between *z* and *x* (see the discussion leading to (6)) becomes $z=(1-A_1^2)x\pm \mathrm {i}\,A_1^2$. Thus $z=\pm \mathrm {i}$ corresponds to $x=\pm \mathrm {i}$. Then taking the limit $x\rightarrow \pm \mathrm {i}$ in the formula (5) for *j*, we see that the ratio $(A_2+D)/A_1$ tends to $\pm \mathrm {i}$ so that $j(Q_{\pm })=P_{\pm }$. $\square$

### Theorem 3

Let $D,E\in {\mathbb {C}}$ be such that $D+2E\ne 0$.

(i) If $1+2DE+4E^2\ne 0$ and $D^2\ne 4$, then the closure ${\bar{X}}(D,E)$ of *X*(*D*, *E*) in $\mathbb {CP}^3$ is a smooth projective curve of genus one. The birational maps *i* and *j* extend to non-trivial involutive automorphisms of ${\bar{X}}(D,E)$, both having fixed points. Their composition $t=j\circ i$ is a non-trivial element of the elliptic curve.
(ii) If $D^2=4$, then ${\bar{X}}(D,E)$ is a rational curve with one node, which a fixed point for both involutions. Their composition is a non-trivial automorphism.
(iii) If $1+2DE+4E^2=0$ and $D^2\ne 4$ then ${\bar{X}}(D,E)$ has two components meeting at two nodes. The involution *i* preserves the components and permutes the nodes, *j* permutes the components and fixes the nodes.

### Proof

The claims of non-triviality of the automorphism follow from Lemma 1: for $D+2E\ne 0$, *j* and *t* map the point at infinity $P_+$ to a finite point $Q_+$.

(i) The first claim follows from the fact that any two-sheeted cover of a smooth rational curve with four simple ramification points is a curve of genus 1. The second claim is a consequence of the fact that any birational map between smooth projective curves is an isomorphism, see [12, Chapter I, Proposition 6.8].

(ii) If $D^2=2$, two of the four ramification points merge at the fixed point $(x,A_1,A_2)=(0,0,-1)$ of both *i* and *j*.

(iii) Here the base curve given by (1) consists of two lines $\ell _\pm$ with equations $A_2-2E=\pm \, \hbox {i}\, A_1$ meeting at the node $(A_1,A_2)=(0,2E)$. There are two ramification points on each line and they are distinct and distinct from the singular point as long as $D^2-4$ (the $A_1$-coordinates of the finite ramification points is $\pm \sqrt{1-D^2/4}$). Thus ${\bar{X}}(D,E)$ is the union of two smooth rational curves $C_{\pm }$, two-sheeted coverings of $\ell _{\pm }$. They meet at two nodes, the preimages of the singular point (0, 2*E*). The sheets and thus the nodes are interchanged by *i*. The nodes represent Kepler conics with foci (0, 0) and (0, 2), and their intersection points with the wall. The conics are symmetric with respect to the reflection at the wall. Therefore,, they cut the wall at straight angles and they are fixed by *j*. Since $D+2E=-1/2E$, the image of $P_+$, which belongs to the component $C_+$, is $(1:A_1:A_2+D:\mathrm {i})$ with $A_1=\mathrm {i}(-D/2-E)$, $A_2=-D/2+E$. Thus $j(P_+)=t(P_+)\in C_-$ and *j* is an isomorphism $C_+\rightarrow C_-$. $\square$

This implies Theorem 2. In particular, in the smooth case (i), there is on ${\bar{X}}(D,E)$ an abelian differential $\lambda$, unique up to normalization, $\varLambda$ is the lattice of integrals of $\lambda$ over closed curves and, for any choice of base point $P_0\in {\bar{X}}(D,E)$ the Abel–Jacobi map $P\mapsto \int _{P_0}^P\lambda$ is a biholomorphic map ${\bar{X}}(D,E)\rightarrow {\mathbb {C}}/\varLambda$.

Under this map, *t* becomes the translation by an element $T\in {\mathbb {C}}/\varLambda$. It can be computed by applying *t* to any point $P\in {\bar{X}}(D,E)$ for instance the point at infinity $P_+=(0:1:\mathrm {i}:0)$ and taking the difference of the images of $Q_+=t(P_+)$ and $P_+$:

$$\begin{aligned} T=\int _{P_+}^{Q_+}\lambda . \end{aligned}$$

More abstractly, ${\bar{X}}(D,E)$ is the fiber of an algebraic family ${\bar{X}}\rightarrow Y={\mathbb {A}}^2\smallsetminus H$ of projective curves of arithmetic genus one over the complement of the line $H=\{D+2E=0\}$ with two sections $P_+,Q_+=t\circ P_+:Y\rightarrow X$. The action of *t* on the fibers is the action of the element of the Jacobian defined by the divisor $P_+-Q_+$.

Here is an explicit parametrization of ${\bar{X}}(D,E)$ by Jacobi elliptic functions. Let *R* be a square root of $R^2=1+2DE+4E^2$. A rational parametrization of the space of solutions of (3) is

9 $$\begin{aligned} A_1=\frac{2\mathrm {i}R s}{1-s^2},\quad A_2=2E+R\frac{1+s^2}{1-s^2}. \end{aligned}$$

and the real points for *D*, *E* real correspond to imaginary *s*.

Then the second equation, in the form (6), is brought to the standard Legendre form

$$\begin{aligned} y^2=(1-s^2)(1-k^2 s^2),\quad k^2=\frac{D+4E-2R}{D+4E+2R}, \end{aligned}$$

by introducing the variable

$$\begin{aligned} y=C^{-1}z(1-s^2),\quad \hbox { where}\quad C^2=(D+2E)(D+4E+2R). \end{aligned}$$

In these variables, the involution *i* is $(s,y)\mapsto (s,-y)$ and a nonzero differential is $\lambda =ds/y$.

The coordinates of $P_+,Q_+$ are

$$\begin{aligned} s(P_+)&=-1,\quad y(P_+)=0,\\ s(Q_+)&=\frac{D+2E+R}{D+2E-R}, \quad y(Q_+)=\frac{-4\mathrm {i}(D+2E)R}{C(D+2E-R)^2}. \end{aligned}$$

The lattice $\varLambda$ is spanned by 4*K* and $2 {\mathrm {i}} K^{\prime }$ where $K,{\mathrm {i}} K^{\prime }$ are the complete elliptic integrals of the first kind

10 $$\begin{aligned} K=\int _{0}^{1}\frac{ds}{\sqrt{(1-s^2)(1-k^2s^2)}}, \quad {\mathrm {i}} K^{\prime }=\int _1^{1/k}\frac{ds}{\sqrt{(1-s^2)(1-k^2s^2)}}. \end{aligned}$$

We then have the parametrization in terms of Jacobi elliptic functions, see, e.g., [5, Chapter 4],

$$\begin{aligned} s={\text {sn}}(u,k), \quad y=\frac{d}{du}{\text {sn}}(u,k) ={\text {cn}}(u,k){\text {dn}}(u,k), \end{aligned}$$

providing an isomorphism ${\mathbb {C}}/\varLambda \rightarrow {\bar{X}}(D,E)$.

Thus we get an explicit uniformization. Instead of the *x*-coordinate *x* of the hitting point, it is convenient to use the variable *z* (proportional to the angular momentum), related to it via (6):

$$\begin{aligned} z= (1-A_1^2)x+A_1(A_2+D). \end{aligned}$$

### Theorem 4

Let (*D*, *E*) satisfy the hypotheses of Theorem 2 and choose a square root of $R^2=1+2ED+4E^2$. Set

$$\begin{aligned} k^2=\frac{D+4E-2R}{D+4E+2R}, \quad s_0=\frac{D+2E+R}{D+2E-R}. \end{aligned}$$

Let $\varLambda ={\mathbb {Z}} 4K+{\mathbb {Z}} 2 {\mathrm {i}} K^{\prime }$ where $K=K(k^2),K^{\prime }=K^{\prime }(k^2)$ are the complete elliptic integrals (10) with Legendre modulus *k*. There is a biholomorphic map $\varphi :{\mathbb {C}}/\varLambda \rightarrow {\bar{X}}(D,E)$ given by

$$\begin{aligned} A_1=2{\mathrm {i}}R \frac{{\text {sn}}u}{{\text {cn}}^2u}, \quad A_2=2E-R+\frac{2R}{{\text {cn}}^2 u}, \quad z=C \frac{{\text {dn}}u}{{\text {cn}}u}. \end{aligned}$$

Here ${\text {sn}},{\text {cn}},{\text {dn}}$ are the classical Jacobi elliptic function with Legendre modulus *k* and $C^2=(D+2E)(D+4E+2R)$. Moreover $\varphi ^{-1}\circ t\circ \varphi$ is the translation $u\mapsto u+T$ by

$$\begin{aligned} T=\int _{-1}^{s_0}\frac{ds}{\sqrt{(1-s^2)(1-k^2 s^2)}}\mod \varLambda , \end{aligned}$$

### Remark 1

There is an ambiguity of sign in the definition of the shift *T* and the choice of square root of *C*. The condition fixing the sign of *T* is that the value of the square root in the integrand at $s=s_0$ is $y(Q_+)$. We will be more precise in the real case below.

## The real locus

So far we considered the problem in the complex domain. Here we want to restrict to the physical region and discuss the quasi-periodicity. For each real values *D*, *E* of the integrals of motion takes place in the set $X_{{\mathbb {R}}}(D,E)$ of solutions of (1), (2).

From the previous section, we know that $X_{{\mathbb {R}}}(D,E)$, with *D*, *E* real satisfying the hypotheses of Theorem 2 has a compactification ${\bar{X}}_{{\mathbb {R}}}(D,E)$ which is the set of real points of a smooth complex projective curve ${\bar{X}}(D,E)$ defined over ${\mathbb {R}}$. The complex conjugation in $\mathbb {CP}^3$ restricts to an antiholomorphic involution $\sigma$ of ${\bar{X}}(D,E)$ and ${\bar{X}}_{{\mathbb {R}}}(D,E)$ is the set of fixed points of $\sigma$. It can be empty or have 1 or 2 connected components. There is one connected components if and only if exactly two ramification points are real. Under the uniformization isomorphism ${\bar{X}}(D,E)\rightarrow {\mathbb {C}}/\varLambda$, $\sigma$ is carried to an antiholomorphic involution of ${\mathbb {C}}$ preserving the lattice $\varLambda$, which we can choose to be $u\mapsto -{\bar{u}}$.

In our case, the ramification points at infinity $(0:1:\pm \mathrm{i}:0)$ are not real, so we have the following two possibilities:

I. $|D|<2$. Exactly two ramification points are real, ${\bar{X}}_{{\mathbb {R}}}(D,E)$, if non-empty, has one connected component. This is the *unipartite case*: the lattice $\varLambda$ is *rhombic* namely it is generated by two periods $\omega ,-{\bar{\omega }}$ which are mapped to each other by the antiholomorphic involution. The real locus ${\mathcal {E}}({\mathbb {R}})$ in ${\mathcal {E}}={\mathbb {C}}/\varLambda$ is isomorphic to the circle group ${\mathbb {R}}/{\mathbb {Z}}$ via
  11 $$\begin{aligned} \theta \mapsto (\omega -{\bar{\omega }})\theta \mod \varLambda , \quad \theta \in \mathbb R/{\mathbb {Z}}. \end{aligned}$$
II. $|D|>2$. No ramification point lies on the real locus, ${\bar{X}}_{{\mathbb {R}}}(D,E)$, if non-empty, has two connected components (the *bipartite case*). The lattice $\varLambda$ is *rectangular*, namely it is generated by a period $\omega _1$ which changes sign under the antiholomorphic involution and a period $\omega _2$ which is fixed by it. The real locus ${\mathcal {E}}({\mathbb {R}})$ in ${\mathbb {C}}/\varLambda$ is isomorphic to the group ${\mathbb {R}}/{\mathbb {Z}}\times {\mathbb {Z}}/2{\mathbb {Z}}$ via
  12 $$\begin{aligned} (\theta ,\epsilon )\mapsto \omega _2\theta +\frac{\omega _1}{2}\epsilon \mod \varLambda , \quad (\theta ,\epsilon )\in {\mathbb {R}}/{\mathbb {Z}}\times {\mathbb {Z}}/2{\mathbb {Z}}. \end{aligned}$$

Thus we have the following result.

### Theorem 5

Let $D,E\in {\mathbb {R}}$ satisfy the hypotheses of Theorem 2 and assume that $X_{{\mathbb {R}}}(D,E)$ is non-empty. Let ${\mathcal {E}}\cong {\mathbb {C}}/\varLambda$ be the elliptic curve associated with *X*(*D*, *E*). Then $t:{\bar{X}}_{{\mathbb {R}}}(D,E)\rightarrow {\bar{X}}_{{\mathbb {R}}}(D,E)$ is the action of an element of the group ${\mathcal {E}}({\mathbb {R}})$ of real points, which is isomorphic to the circle group $S^1$ if $|D|<2$ and to $S^1\times {\mathbb {Z}}/2{\mathbb {Z}}$ if $|D|>2$.

We next examine the condition for the real locus to be non-empty, give an explicit parametrization of $X_{{\mathbb {R}}}(D,E)$, and identify the element of ${\mathcal {E}}({\mathbb {R}})$ by which *t* acts.

We first notice that we have a bijection $X_{{\mathbb {R}}}(D,E)\rightarrow X_{{\mathbb {R}}}(-D,-E)$ sending $(A_1,A_2,x)$ to $(-A_1,-A_2,x)$. This bijection commutes with the involutions and changes the sign of $L^2=D+2A_2$. We can assume that $D+2E>0$, which is the physical region where the angular momentum is real. Indeed it follows from (3) and the inequality $A_1^2+(A_2+D)^2\ge 1$, guaranteeing the existence of real solutions *x* of (2) that $(D+2E)(D+2A_2)\ge 0$ and $L^2=D+2A_2$ is nonnegative on $X_{{\mathbb {R}}}(D,E)$ only if $D+2E>0$.

A necessary condition for $X_{{\mathbb {R}}}(D,E)$ to be non-empty is that $1+2ED+4E^2\ge 0$, see (3). It is then convenient to introduce the radius as the nonnegative square root

$$\begin{aligned} R=\sqrt{1+2ED+4E^2}. \end{aligned}$$

### Lemma 2

Assume $D,E\in {\mathbb {R}}$ satisfy the inequalities $R>0$, $D+2E>0$. Then $X_{{\mathbb {R}}}(D,E)$ is non-empty if and only if $D+4E+2R>0$.

### Proof

The configuration space is defined by the two Eqs. (1), (2). The second equation has real solutions for *x* if and only if the discriminant $A_1^2+(A_2+D)^2-1$ is nonnegative. Thus $X_{{\mathbb {R}}}(D,E)$ is non-empty if and only if there exists $(A_1,A_2)\in {\mathbb {R}}^2$ such that

$$\begin{aligned} A_1^2+(A_2-2E)^2&=R^2,\\ A_1^2+(A_2+D)^2&\ge 1. \end{aligned}$$

Taking the difference we can replace the inequality by $(D+2E)(D+2A_2)\ge 0$ or $A_2\ge -D/2$. Given $A_2$ satisfying this inequality, we find $A_1$ if and only if $(A_2-2E)^2\le R^2$. These two inequalities for $A_2$ can be simultaneously satisfied if and only if $D+4E+2R\ge 0$. We still have to show that this inequality cannot be an equality. If $D+4E=-2R$, then taking squares gives

$D^2=4$, which is excluded by the assumption of Theorem 4. $\square$

**Fig. 3:**
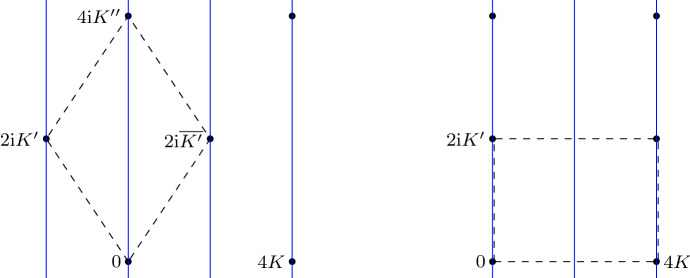
The lattice $\varLambda$ of periods in the unipartite case (left) and the bipartite case (right). The vertical lines project to the real locus on ${\mathbb {C}}/\varLambda$. The dashed line indicate a fundamental domain

In terms of the Legendre coordinates, the real points correspond to imaginary *s* and real *y*. The involution is $s\mapsto -{\bar{s}}$, $y\mapsto {\bar{y}}$. Thus $u=\int ds/y$ is mapped to $-{\bar{u}}$ under the antiholomorphic involution. The periods $\omega ,\omega _1,\omega _2$ can be expressed in terms of complete elliptic integrals as follows; see Fig. 3.

### Lemma 3

Let *K*, $K^{\prime }$ be defined for $0<k<1$ to be real and positive. Extend the definition to the case of *k* in the upper half plane by analytic continuation.

I. If $|D|<2$ then $k^2<0$. Let $k=\mathrm {i}\ell$ with $\ell >0$. Then *K* is real and $2\mathrm {i}K^{\prime }=-2K+2\mathrm {i}K^{\prime \prime }$ where
  $$\begin{aligned} K^{\prime \prime }=\int _{0}^{1/\ell }\frac{dv}{\sqrt{(1+v^2)(1-\ell ^2v^2)}} \in {\mathbb {R}}_{>0}. \end{aligned}$$
  Thus $\varLambda$ is spanned by $\omega =2K+2\mathrm {i}K^{\prime \prime }$, ${\bar{\omega }}=-2K+2\mathrm {i}K^{\prime \prime }$.
II. If $|D|>2$ then $0<k^2<1$ and $\varLambda$ is spanned by $\omega _1=4K$ and $\omega _2=2\mathrm {i}K^{\prime }$.

### Proof

The denominator of $k^2$ is positive by Lemma 2. With the identity $(D+4E+2R)(D+4E-2R)=D^2-4$, we see that the numerator is positive if and only if $D^4>4$. In this case, the numerator is smaller than the denominator since $R>0$. Thus $k^2<0$ if $D^2<4$ and $0<k^2<1$ if $D^2>4$. Let $|D|<2$. Then *K* is positive and $\mathrm {i}K^{\prime }$ is given by analytic continuation for $k=\mathrm {i}\ell$ in the upper half-plane. The integration path from 1 to $1/k=-\mathrm {i}/\ell$ can be deformed to a path going from 1 to 0 and then continuing to $-\mathrm{i}/\ell$ along the imaginary axis. Thus $\mathrm {i}K^{\prime }=-K+\mathrm {i}\int _0^{1/\ell }dv/\sqrt{(1+v^2)(1-\ell ^2v^2)}$. In the case II, no analytic continuation is needed. $\square$

**Fig. 4:**
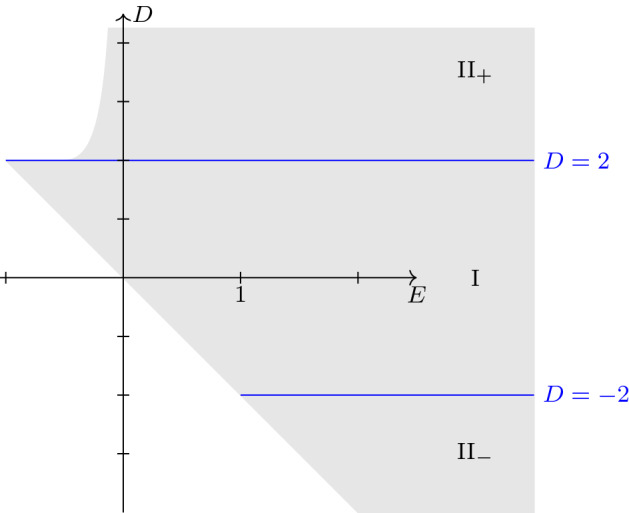
The physical domain of parameters, with the three regions delimited by the singular locus $D=\pm 2$. In the region I, the real locus of the elliptic curve is isomorphic to $S^1$. In the regions II${}_\pm$, it is isomorphic to $S^1\times {\mathbb {Z}}/2 {\mathbb {Z}}$ and the dynamics is given by the translation of an element in the connected component of the identity (case II${}_-$) or the other component (case II${}_+$)

### Theorem 6

Assume $(D,E)\in {\mathbb {R}}^2$ satisfies the hypotheses of Theorem 2 and the real angular momentum condition $D+2E>0$. Then $X_{{\mathbb {R}}}(D,E)$ is non-empty in the following three cases (see Fig. 4).

I: Let $-2<D<2$, $D+2E>0$. In this case $k^2<0$, $|s_0|>1$. Write $k=\mathrm {i}\ell$. Then there is a diffeomorphism $\varphi :{\mathbb {R}}/{\mathbb {Z}}\rightarrow X_{{\mathbb {R}}}(D,E)$ such that $\varphi ^{-1}\circ t\circ \varphi (\theta )=\theta +\alpha$ is the shift by
  $$\begin{aligned} \alpha =\alpha (D,E)=- \frac{1}{4K^{\prime \prime }}\int _{-1}^{1/s_0}\frac{ds}{\sqrt{(1-s^2)(\ell ^2+s^2)}}. \end{aligned}$$
II$${}_+$$
Let $D>2$, $E>-1/2$, $1+2ED+4E^2>0$. In this case, $0<k^2<1$ and we have $1<s_0<1/k$ where *k* is the positive square root. Then there is a diffeomorphism $\varphi :{\mathbb {R}}/{\mathbb {Z}}\times {\mathbb {Z}}/2{\mathbb {Z}}\rightarrow X_{{\mathbb {R}}}(D,E)$ such that $\varphi ^{-1}\circ t\circ \varphi (\theta ,\epsilon )=(\theta +\alpha ,\epsilon +{\bar{1}})$ is the composition of the shift by
  $$\begin{aligned} \alpha =\alpha (D,E)=\frac{1}{2K^{\prime }}\int _{1}^{s_0} \frac{ds}{\sqrt{(s^2-1)(1-k^2s^2)}} \end{aligned}$$
  and the generator ${\bar{1}}$ of the subgroup ${\mathbb {Z}}/2{\mathbb {Z}}$.
II$${}_-$$
Let $D<-2$, $D+2E>0$. In this case, $0<k<1$ and $-1/k<s_0<-1$. There is a diffeomorphism $\varphi :{\mathbb {R}}/{\mathbb {Z}}\times {\mathbb {Z}}/2{\mathbb {Z}}\rightarrow X_{{\mathbb {R}}}(D,E)$ such that $\varphi ^{-1}\circ t\circ \varphi (\theta ,\epsilon )=(\theta +\alpha ,\epsilon )$ is the shift by
  $$\begin{aligned} \alpha =\alpha (D,E)=-\frac{1}{2K^{\prime }}\int _{s_0}^{-1} \frac{ds}{\sqrt{(s^2-1)(1-k^2s^2)}}. \end{aligned}$$

In these formulas,, we take the positive square root in the integrands. In all cases, $C^2=(D+2E)(D+4E+2R)>0$. The map $\varphi$ is the parametrization of Theorem 4 with $C>0$ and with $u=4\mathrm {i}K^{\prime \prime }\theta$ in case I and $u=2\mathrm {i}K^{\prime }\theta +\epsilon 2K$ in case II${}_\pm$. Moreover, for any *E*, the derivative $\partial \alpha (D,E)/\partial D$ does not vanish on a dense open set.

### Proof

By Lemma 2, $X_{{\mathbb {R}}}(D,E)$ is non-empty if and only if

13 $$\begin{aligned} D+4E+2R>0. \end{aligned}$$

This condition is empty if $E\ge 0$ since the left-hand side is the sum of $D+2E>0$, $2E\ge 0$, $2R>0$. So let $E<0$. Eliminating *D* yields the condition

$$\begin{aligned} \frac{(R+2E+1)(R+2E-1)}{2E}\ge 0. \end{aligned}$$

Since $R^2=1+(D+2E)2E<1$, the second factor in the numerator is negative, so the condition reduces to $R+2E+1\ge 0$. This condition is empty if $E\ge -1/2$. If $E<-1/2$, it can be written as $R^2> (2E+1)^2$, which is equivalent to $2ED > 4E$, i.e., $D<2$. We conclude that (13) is automatically satisfied if $D<2$ (under the assumption that $D+2E>0$), and requires $E>-1/2$ if $D>2$. In all cases, $C^2=(D+2E)(D+4E+2R)>0$.

Similar elementary considerations lead to the following inequalities for $s_0$.

I.: Let $-2<D<2$, $E>-D/2$. In this case $k^2<0$, $|s_0|>1$. Write $k=\mathrm {i}\ell$. The automorphism *t* acts on ${\mathbb {C}}/\varLambda$ by translation by the elliptic integral *T* of Theorem 4. Since we take $u=4\mathrm {i}K^{\prime \prime }\theta$, the shift of $\theta$ is $T/(4\mathrm {i}K^{\prime \prime })$. The only subtlety is to fix the sign. The sign of the square root in the integrand is determined by the condition that its value at $s=s_0$ is $y(Q_+)$ which is negative imaginary (see Remark 1). Thus we can write *T* as
  $$\begin{aligned} T=\mathrm {i}\int _{-1}^{s_0}\frac{ds}{\sqrt{(s^2-1)(1+\ell ^2s^2)}}. \end{aligned}$$
  where we integrate over a path connecting $-1$ and $s_0$ along the negative real axis (and going through the point at infinity if $s_0$ is positive). The square root is taken to be positive. A more convenient expression is obtained by the variable substitution $s\mapsto 1/s$ since $1/s_0$ is then in the interval $(-1,1)$ and the integral is over a finite interval.
II$${}_+$$.
Let $D>2$, $1+2 DE+4E^2>0$. In this case we can take $0<k<1$ and $1<s_0<1/k$ The integrand in the elliptic integral *T* is real in the interval $(-1,1)$ and imaginary in the interval $[1,s_0]$. The integral over $(-1,1)$ is 2*K* (the sign does not matter here since $-2K\equiv 2K\mod \varLambda$) and the sign of the integral from 1 to $s_0$ is fixed as above by the condition that the value of the square root at the end point $s_0$ is negative imaginary. We get
  $$\begin{aligned} T=2K+\mathrm {i}\int _{1}^{s_0}\frac{ds}{\sqrt{(s^2-1)(1-k^2 s^2)}}. \end{aligned}$$
II$${}_-$$.
Let $D<-2$, $E>-D/2$. In this case $0<k<1$ and $-1/k<s_0<-1$. This case is treated similarly to the previous one.

Finally, the fact that $\alpha (D,E)$ has nonzero derivative follows by looking at the limit $D\rightarrow -2E$. In this limit, $s_0$ tends to $-1$, so $\alpha (D,E)$, which does not vanish being given by an integral of a positive function, tends to 0. The parameter $k^2$ of the elliptic curve tends to $(E-1)/(E+1)$ which is never 1, so that $K^{\prime \prime }$ and $K^{\prime }$ have a finite nonzero limit. Thus $D\mapsto \alpha (D,E)$ is a real analytic function which is non-constant on some interval. It has therefore only isolated critical points. $\square$

**Fig. 5:**
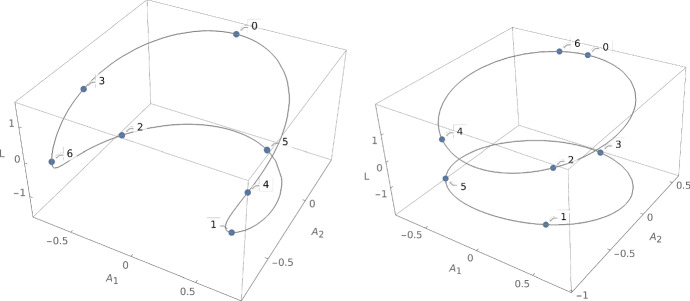
The level sets $X_{{\mathbb {R}}}(1.5,-0.2)$ (Type I, left) and $X_{{\mathbb {R}}}(2.5,-0.1)$ (Type II${}_+$, right) in the coordinates $A_1,A_2$ of the Laplace–Runge–Lenz vector and the angular momentum *L*, with six iterations of *t*

This parametrization allows us to plot the level sets and the orbits. We do this in Fig. 5 using the Laplace–Runge–Lenz vector and the angular momentum $L=z(D+2E)^{-\frac{1}{2}}$ as coordinates.

### Remark 2

In the negative energy case $E<0$, the motion takes place in a compact region of the plane so $X_{{\mathbb {R}}}(D,E)={\bar{X}}_{{\mathbb {R}}}(D,E)$. If $E>0$, one has collision points at infinity corresponding to hyperbolae with an asymptote parallel to the wall.

## Footnotes

1 Unlike certain name-dropping titles.

2 In [7], the authors consider the case of a particle of mass $m=1$ in a potential with coupling constant $\kappa =2\alpha$ and energy $E=A/2$; *D* is called *R* there. The radius of the circle on which the midpoint of the ellipse moves (*R*/2|*E*|*m* in our notation) is denoted by $R_0$ in loc. cit.
